# Medical Items

**Published:** 1896-11

**Authors:** 


### *MEDICAL ITEMS.*

---

Sir George Humphry, Professor of Surgery at the University of Cambridge, and Sir J. Eric Erichsen, ex-President of the Royal College of Surgeons of England, etc., whose writings on surgical subject are so well known, have both died recently.

---

Personal.—Dr. Wm. Curtiss Bailey, who was in Atlanta last winter and lectured at the Atlanta Medical College, is now Medical Director of the Las Vegas Hot Springs Sanitarium, New Mexico. The Institution will be conducted especially for those who are afflicted with tuberculosis, chiefly in the earlier stages. The situation and management of the Sanitarium are excellent for the purpose.

---

The annual meeting of the Clinical Society of Maryland was held in the Hall of the Medical and Chirurgical Faculty, 847 North Eutaw Street. The President, Dr. J. M. Hundley, was in the chair.

Reports of the officers were received and the president expressed his thanks to them, especially to the Executive Committee, for their assistance in making the past such a successful year.

The election resulted in naming the following gentlemen to serve for the ensuing year, viz.:

President, Dr. S. K. Merrick; Vice-President, Dr. W. D. Booker; Recording Secretary, Dr. H. O. Reik; Corresponding Secretary, Dr. W. G. Townsend; Treasurer, Dr. W. J. Todd; Member Finance Committee, Dr. J. M. Hundley; Executive Committee, Dr. J. W. Lord, Chairman, Dr. W. B. Canfield, Dr. T. P. McCormick.

Who is this? We learn from a northern exchange that the following attractive advertisement is carried on the stationery of a "Georgia graduate":

After 5 days return to : : :

Dr. ——— ———

Graduate from Medical College, Atlanta, Georgia.

SPECIALTIES:—Obstetrics and results; Disease of Females, Chronic or Acute. Cancers, Hemorrhoids (piles). Cure guaranteed if instructions are carried out. Very little pain attached to remedy, without legation or knife. Tumors and Wens cured without the use of knife. Good references given if called upon. I also have a genuine Madstone, has been successful preventing hydrophobia after being bitten by Rabid dogs, etc.

Charges reasonable. The doctor will visit his patients at their private rooms when called upon, and can be found by inquiry at his office at

. . . . . . . . . . . . . . . . . . . . . . . . . . . . . . . . . . . . . . . . . . .

I also have some very desirable property in the town of ——— for sale.

THE following appears in the *Southwestern Medical and Surgical Reporter:*

THE MEDICAL MAID.

*The Professor* (loquitur).

As I gaed to meet my class,
  A winsome quean came after me;
Quo' she, " Sir, wad ye gi'e a lass
  Prelections on Anatomy?"
I spiered was there nae work at hame
  Micht suit her mair becomingly,
For delvin' in a corpse's wame
  Was neither sweet nor womanly.

But deil may care, she said her share
  Was not domestic drudgery;
An' threapit that her mission here
  Was medicine and surgery;
Nae longer she'd her cawnle hide
  Aneath a bushel, scomfishin',
Since woman noo wi' rapid stride
  Her function was accomplishin'.

Afore her powers o' speech I fell,
  She fairly got command o' me,
I felt I wasna just mysel'
  An' she'd the upper hand o' me.
Wi' smiles sae sweet, an' words sae fair,
  She made I watna what o' me;
Says I: " My woman, fleech nae mair:
  I'll drill ye in Anatomy,"

I thocht I did but what was richt,
  Tae treat her wi' urbanity;
My state, hooever, since that nicht,
  Has bordered on insanity.
I'm clean dumfoundered what to say,
  What pairts tae gi'e, whaur she's tae be;
For decency, I think I'll hae
  Her demonstrations privately.

My best cadaver she at aince
  Bespoke in its integrity;
It was a male; I cudna but
  Admire her intrepidity;
When I suggested something mair
  Consistent wi' propriety,
An aince she said she wadna be
  Restrickit in variety.

Frae day till day I've thocht the jade
  Wad drive the senses oot o' me,
Wi' spierin' whiles aboot sic things
  As did pit me aboot a wee;
An' aye she'd houp I'd not forget,
  Wi' want of generosity,
'T was science set her there, and not
  A morbid curiosity.

My words oot o' respect for her
  I've trimmed wi' ingenuity;
An aince she said my language was
  Devoid o' perspicuity.
She disna gi'e a single flea
  For feminine timidity,
Demandin' baith to hear and see
  The plain facts wi' lucidity.

It's no mysel' alane she skeers
  Frae speakin' wi' security:
Ilk bashfu' lecturer she hears,
  Takes refuge in obscurity;
Oor houdies a' she pits till shame,
  For wi' superiority,
It speaks aboot the female frame
  On sic direct authority.

Ilk jurisprudence lecturer
  Complains o' his predicament;
He canna thole tae speak tae her
  On horrors that suld mak' her faint.
It's kittle no tae feel confused,
  Or tell her wi' facility
O' things that micht mak' her amazed,
  If that's a possibility.

The surgical professors say
  It wad be sae incongruous
O' certain ills tae speak, that they
  Maun modify their syllabus,
A class o' matrons they micht face,
  O' strict respectability,
But frisky kimmers in their place
  Tells on their equanimity.

The folk outside bid us gi'e in,
  Sin we're in the minority;
They haud oor case no worth a prin
  An' hers tae hae priority.
I was a gowk tae tak' her in,
  For now she'll no gang oot again;
Man's first mishap cam' by a lass—
  My days hae been cut short by ane.

